# Microglial VPS35 deficiency impairs Aβ phagocytosis and Aβ-induced disease-associated microglia, and enhances Aβ associated pathology

**DOI:** 10.1186/s12974-022-02422-0

**Published:** 2022-03-02

**Authors:** Xiao Ren, Lingling Yao, YongGang Wang, Lin Mei, Wen-Cheng Xiong

**Affiliations:** 1 Department of Neurosciences, Case Western Reserve University, Cleveland, OH USA; 2 Beijing Tiantan Hospital, Capital Medical University, No.119, S 4th Ring W Rd, Fengtai District, Beijing, 100070 China; 3 Department of Neurology, Renji Hospital, Shanghai Jiao Tong University, Shanghai, China

**Keywords:** Alzheimer’s disease, VPS35, Retromer, Aβ, Microglia, DAM

## Abstract

**Background:**

Vacuolar sorting protein 35 (VPS35), a key component of the retromer, plays an essential role in selectively retrieval of transmembrane proteins from endosomes to trans-Golgi networks. Dysfunctional retromer is a risk factor for neurodegenerative disorders, including Alzheimer’s disease (AD). Microglial VPS35 deficiency is found in AD patients’ brain; however, it remains unclear if and how microglial VPS35-loss contributes to AD development.

**Methods:**

We used mice with VPS35 cKO (conditional knockout) in microglial cells in 5XFAD, an AD mouse model. The AD related brain pathology (Aβ and glial activation), behavior, and phagocytosis of Aβ were accessed by a combination of immunofluorescence staining analyses and neurological behavior tests.

**Results:**

A decrease in learning and memory function, but increases in insoluble, fibrillar, and plaques of β-amyloids (Aβ), dystrophic neurites, and reactive astrocytes are observed in microglial VPS35 deficient 5XFAD mice. Further examining microglial phenotype demonstrates necessity of microglial VPS35 in disease-associated microglia (DAM) development and microglial uptake of Aβ, revealing a tight association of microglial Aβ uptake with DAM development.

**Conclusions:**

Together, these results uncovered a mechanism by which microglial VPS35-deficiency precipitates AD pathology in 5XFAD mice likely by impairing DAM development and DAM mediated Aβ uptake and clearance, and thus accelerating the cognition decline.

**Supplementary Information:**

The online version contains supplementary material available at 10.1186/s12974-022-02422-0.

## Background

Alzheimer’s disease (AD) is a progressive neurodegenerative disorder characterized by cognitive dysfunction and memory loss [1, 2]. Pathological hallmarks of AD patients include the amyloid-beta (Aβ) plaque deposition, neurofibrillary tangles formation, neuron loss, dystrophic neurites, and the gliosis [3].

Recent GWAS studies on AD patients have identified many AD risk genes. Interestingly, majority AD risk genes are abundantly and selectively expressed in immune cells, such as microglial cells, the macrophage residents in the brain [4]. Thus, it is of considerable interest to investigate functions of microglia in AD pathogenesis. Numerous studies have suggested a protective role of microglia in restraining Aβ accumulation and incidence of AD [5]. On the other hand, microglial activation in AD also has a detrimental functions, such as engulfing neuronal synapses and secreting inflammatory factors [6]. Recent single cell RNA-seq analysis reveals subtypes of microglia in response to stimulation through different pathways, including M1, M2, and disease associated microglia (DAM) [7]. However, how these different subtypes of microglia are formed and how they contribute to AD development remain elusive.

The retromer complex contains two major subcomplexes, the cargo-selective subcomplex and membrane deformation subcomplex. The vacuolar protein sorting 35 (VPS35) was a key component in the cargo-selective subcomplex that sorts cargos (membrane proteins) into tubules for retrieval to the Golgi apparatus [8–10]. Dysfunctional of retromer is believed to be a risk factor for multiple neurodegenerative disorders, including AD and Parkinson’s disease (PD) [11–13]. VPS35/retromer deficiency is found in the hippocampus of AD patients [14]. Vps35 haploinsufficiency in Tg2576 AD animal model increases AD neuropathology [9]. It is known that VPS35/retromer is widely expressed, including neurons and microglial cells [15, 16]. We have previously demonstrated that neuronal VPS35 deficiency results in neurodegenerative pathology in developing mouse brain [17, 18] and microglial VPS35-loss induces hippocampal microglial cell activation, impairs adult hippocampal neurogenesis [15], but decreases ischemic stroke-induced damage in the cortex [19]. Notice that microglial VPS35 is reduced in microglia derived from AD patients’ brain [20]. However, if and how microglial VPS35 deficiency contributes to AD development remain largely unknown.

Here, we provide evidence for microglial VPS35-loss to expedite AD pathology in 5XFAD mouse model. Microglial VPS35 deficient 5XFAD mice (VPS35^CX3CR1−Cre^; 5XFAD) exhibit increases in insoluble, fibrillar, and plaques of β-amyloids (Aβ), dystrophic neurites, and reactive astrocytes, and decrease in learning and memory function. Examination of microglial phenotypes in 5XFAD brain demonstrates that microglial VPS35 is necessary for DAM formation. Evaluation of microglial uptake of injected Aβ in microglial VPS35 deficient mice reveals critical roles of microglial VPS35 not only in Aβ phagocytosis, but also in Aβ-induced DAM formation. These results thus demonstrate microglial VPS35 deficiency’s contribution to 5XFAD pathology, uncovering a mechanism for VPS35 deficiency in AD development.

## Methods

### Animals

*VPS35*^*flox/flox*^ (*VPS35*^*f/f*^) mice were generated as previously described [15]. *CX3CR1*^*Cre−ER*^ and 5xFAD [B6SJL-Tg (APPSwFlLon, PSEN1 × M146L × L286V) 6799Vas/Mmjax] mice were purchased from the Jackson Laboratory (Stock No: 021160, MMRRC Stock No: 34840-JAX, respectively). Note that 5xFAD mice express mutant human amyloid beta precursor protein (APP) and human presenilin 1 (PSEN1), with a total of five mutations including the Swedish [K670N/M671L], Florida [I716V], and London [V717I] in APP and M146L and L286V in PSEN1 under the control of Thy1-promoter [21]. *VPS35*^*f/f*^ mice were crossed with *CX3CR1*^*Cre−ER*^ mice as described previously [15, 19], and then crossed with 5xFAD to generated *VPS35*^*f/f*^:*CX3CR1*^*Cre−ER*^*:5xFAD*. All mice were maintained in C57BL/6 strain background for > 6 generations. All mice were group-housed with no more than 5 per cage under a room with 12 h light/dark cycle with water and standard rodent chow diet. All experiments with animals were approved by the Institutional Animal Care and Use Committee at Case Western Reserve University.

### Experimental design and statistical analysis

At postnatal day of 15, 45, 75, mice were injected with100 mg/kg of tamoxifen (Sigma Millipore, catalog #T5648) dissolved in corn oil (Sigma Millipore, catalog #C8267) intraperitoneally for 3 times consecutive in 1-day intervals at each injection day (labeled as VPS35^CX3CR1^:5xFAD). Unless indicated specifically, the age-matched control mice received same dose corn oil as vehicle by injection (labeled as Ctrl:5xFAD). All results were confirmed with tamoxifen injection in *VPS35*^*f/f*^*:5xFAD* and *CX3CR1*^*Cre−ER*^*:5xFAD* to eliminate the potential effects of tamoxifen.

All data were presented as mean ± SD. 5 or more brain slices from 3 or more mice in each group were used for immunohistochemical analyses. > 6 mice in each group were assigned for behavioral test. All immunofluorescence staining data were quantified by ImageJ software. Statistical analyses were performed using Prism 7 (GraphPad Software). For two independent data comparisons, unpaired Student’s *t* test was used to determine statically significance. The test was considered significant when *P* < 0.05.

### Tissue processing and immunostaining

Mice were anesthetized by 3% isoflurane and perfused with phosphate-buffered saline (PBS, 0.01 M, pH = 7.4), then followed by 4% paraformaldehyde (PFA), and brains were post-fix overnight with 4% PFA. Brain tissues were sectioned into 30 μm-thick free-floating coronal or sagittal sections with different purposes using a vibratome (Leica VT1000S). All brain slices were sequentially collected and stored at -20 °C in cryoprotectant solution (FD Section Storage Solution) for further staining.

Free-floating sections were washed in PBS (3–5 min, 3 times) and incubated in blocking buffer (0.03% Triton X-100, and 2% donkey serum in PBS) for 30 min. Then the slices were incubated in primary antibody for overnight at 4 °C, washed 3 time in PBS in the next day, followed by incubating in secondary antibodies for 2 h at room temperature. Primary antibodies used were as follows: anti-VPS35, generated by Xiong lab as previously described [8]; anti-Iba1, ab178846, Abcam; anti-Iba1, ab5076, Abcam; anti-OC, AB2286, Sigma-Aldrich; anti-6E10, 803015, Biolegend; anti-ATG9A, ab108338, Abcam; anti-RTN3, 12055-2-AP, Thermofisher; anti-GFAP, 12389, Cell Signaling; anti-APOE, K74180B, Menidian Life Science; anti-TMEM119, ab209064, Abcam; anti-LPL, ab21356, Abcam; anti-Trem2, MAB2056, Abnova; anti-Clec7A, mabg-mdect, InvivoGen.

### Aβ deposition assay

Thioflavin S (Thio-s) staining was used for Aβ deposition assay as previously described with mini modification. Every fifth tissue section of five consecutive sagittal sections was collected for Immunofluorescence staining. The brain slices were washed with PBS for 3 times and incubated in 0.1% Thio-S solution for 10 min. Then the brain slices washed with a series of graded EtOH as follows: 95% EtOH for 3 min, 80% EtOH for 3 min. Finally, the brain slices washed by PBS for 3 times. All the brain slices were captured on a BZX slider scanner. The images were converted and adjusted to the same threshold to increase signal-to-noise ratio. Five sections per mice from ctrl:5xFAD (*n* = 10) and VPS35^CX3CR1^:5xFAD (*n* = 9) were quantified. In brief, the area of cortex (CTX), hippocampus (HIP), subiculum (SUB) and thalamus (TH) were outlined and quantified. The plaque density (plaque^+^ area /5.5 µm^2^) was quantified by the National Institutes of Health ImageJ software.

### Image analysis

For immunostaining analyses, the brain slices were collected every 5th brain slice from bregma to lambda in each mouse, and 3 mice from each group were quantified. For OC/6E10 quantification, the representative field in the somatosensory cortex and hippocampus were selected in matched area. The ROI (region of interest) area was defined as 638.9 μm × 638.9 μm and randomly selected by a blinded observer. The OC density was calculated as: Area_OC_/Area_ROI_ × 100%. The plaque diffuseness index was calculated by (Area_6E10_ − Area_Thio-S_)/ Area_6E10_, as described previously [22]. For quantification of Aβ-associated dystrophic neurites, the matched area in cortex and hippocampus were picked as previously described [23]. The selected areas containing the Aβ plaque core and the surrounding 50 μm in diameter were quantified. The percentage burden (% of pixels with positive staining for ATG9A and RTN3) were quantified in the Aβ-associated area, as described previously [22]. For Aβ associated microglia quantification, two representative brain regions, the somatosensory cortex and hippocampus, were chosen from each brain slice. The Aβ associated area was defined by a plaque centered circle within 50 μm in diameter, and the number of microglia within the circle was quantified by ImageJ. For disease associated microglia (DAM) quantification, the Aβ associated area and Aβ un-associated area were randomly selected from the matched regions in the somatosensory cortex, and the fluorescence of DAM marker(s) co-stained with Iba1 was quantified and normalized with the intensity of control group. For Aβ phagocytosis, the fluorescence intensity of Aβ, LPL, and Clec7a were quantified in injected site and un-injected site with match area in somatosensory cortex. For these analyses, five slices from medial to lateral in each mouse, and 3–6 mice from each group were quantified with ImageJ by a blind observer.

### Elisa assay

The brain homogenization from mouse cortex and hippocampus was obtained as previously described [24]. The frozen tissue (PBS buffer) was homogenized with Dounce homogenizers until no visible pieces were seen; centrifuged (12,000 g, 4 °C, 30 min); and the supernatants were collected for soluble Aβ Elisa analysis. The insoluble fraction by PBS buffer were further solubilized by adding 5 M Guanidine buffer at the same ratio and homogenized by sonication. The homogenates were blended at room temperature for 6 h, and the supernatants were collected for insoluble Aβ Elisa analysis. The concentrations of β-amyloids were measured by ELISA kits for human Aβ42 (Invitrogen, catalog # KHB3441), human Aβ40 (Invitrogen, catalog # KHB3481), and mouse Aβ40 (Invitrogen, Catalog # KMB3481), respectively, following the manufacture instruction.

### Phagocytosis assay

Aβ42 (Bachem, catalog #H-1368) was purchased from AnaSpec and the pHrodo Red Zymosan Bioparticles was purchased from ThermoFisher (Catalog # P35364). The phagocytosis assay was performed as described previously [25]. Briefly, Aβ42 peptide was dissolved in 1% NH4OH at 1 mg/ml and sonicated. Lyophilized Aβ42 was further dissolved in water, filtered (0.22 mm), and incubated at 37 °C for 24 h before use. Mice were anesthetized with isoflurane, and they were mounted in a stereotaxic apparatus and injected with 1 μl of aged Aβ42 and same volume of Phred beads slowly (0.1 μl/min) into the cortex of control and VPS35^CX3CR1^ mice according to mouse brain atlas [26, 27]. The coordinates relative to bregma for cortex injection was caudal: − 2.06 mm; lateral: +/− 1.5 mm; ventral: − 0.8 mm. The needle of microinjector was left at the injection site for 5 min to enable its diffusion, and slowly withdraw. The mice were sacrificed on day 7 after the injection.

### Behavior tests

All male mice at the age of 3-MO (month) were used for behavior tests. Mice were transferred to the behavior test room 2 h before onset of behavior test to acclimate the environment. Before the onset of each trial, behavioral areas were sprayed with 75% ethanol. Unless otherwise noted, all behavioral trials were recorded using an overhead camera and analyzed by Etho Vision software (Etho Vision, Noldus). Mice were assigned and data quantified in double-blind method.

For open field test, each mouse was introduced into a chamber (50 × 50 × 20 cm, WxDxH) and allowed free move for 6 min. Total traveled distance and time in the inner zone were quantified. For depressive-like behavioral paradigms, force swimming test (FST), tail suspension test (TST) and sucrose preference test (SPT) were performed as previously described [28, 29]. Mice were recorded 6 min and quantified the mobility time in FST and TST, for the sucrose preference test, the mice were individually habituated to drink 2% (wt/vol) sucrose solution (dissolved in water) for 3 days, then mice were given access to the two pre-weighed bottles, one containing water and the other containing 2% sucrose solution. Bottle positions were changed every day and water and sucrose solution consumption was assessed daily for 4 days. The sucrose preference was quantified by the ratio of sucrose consumed over total solution consumed was used for measuring the sucrose preference. Y-Maze test, as previously described [30], each mouse was placed at the central of three opaque plastic arms and allowed to freely explore the three arms for 8 min. Total arm of entries and spontaneous alternation were quantified. For the Morris water maze (MWM), a circular water tank (diameter of 120 cm) fulfilled with water. The maze was equally divided into 4 quadrants, and one quadrant contain a platform with diameter of 10 cm as the escape platform. Then nontoxic white powder paints were added to the water to make the surface opaque to hide the escape platform. Mice were trained 4 trials per day with 20 min interval for 5 consecutive days. Mice were allowed to free search the escape platform for 60 s, at which point the experimenter would guide the animal to the escape platform if necessary, and the time latency will be quantified as 60 s. On the 6th day, the escape platform will be removed, and mice will have 60 s to swim. The swimming trajectories and the time spend in target quadrant will be recorded and quantified.

## Results

### Increased Aβ in in-soluble, oligomer, and plaque forms in microglial VPS35 deficient 5XFAD brain

To investigate if microglial VPS35 deficiency contributes to the AD pathology, *VPS35*^*CX3CR1*^*:5xFAD* mice were generated by crossing *VPS35*^*f/f*^ mice with *CX3CR1*^*Cre−ER*^ and 5xFAD mice (Additional file 1: Fig. S1a). The *VPS35*^*CX3CR1*^*:5xFAD* mice were injected with tamoxifen or vehicle (as control) starting at age of P15 for three times as indicated in Additional file 1: Fig. S1b. At P90, mice were subjected to various tests, including brain pathology examinations and behavior assessment (Additional file 1: Fig. S1b). Notice that VPS35 was specifically knock out in the Iba1^+^ microglia cells in *VPS35*^*CX3CR1*^*:5xFAD* brain, as compared with that in Ctrl:5xFAD mice (Additional file 1: Fig. S1c, d), verified the mouse identify. No difference in their body weights was detected between two group of mice at the age of 3 MO (Additional file 1: Fig. S1e).

**Fig. 1:**
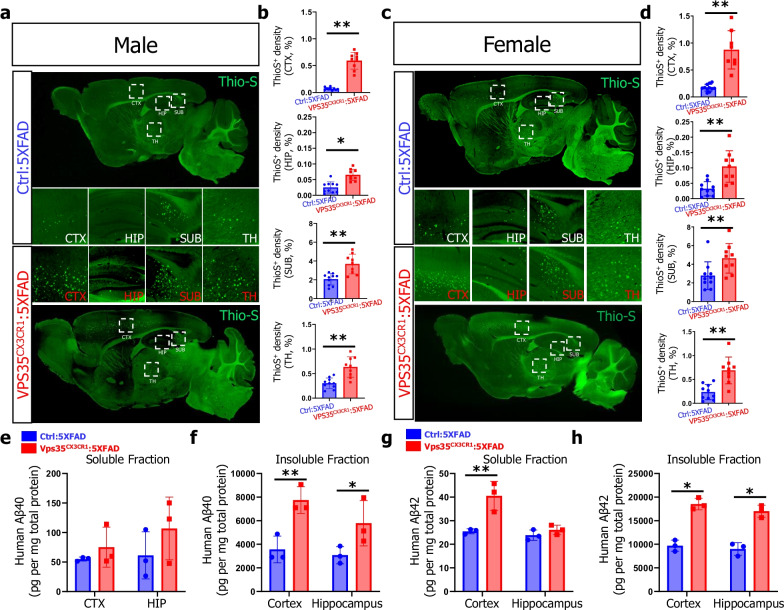
Increased Aβ levels and Aβ plaques in microglial VPS35 deficient 5XFAD brain. **a–d** Increased Aβ plaque depositions in 3-mo VPS35^CX3CR1^:5XFAD male (**a**, **b**) and female (**c**, **d**) brains. High-magnification images in cortex (CTX), hippocampus (HIP), subiculum (SUB), and thalamus (TH), marked by dashed squares, were shown. Quantification data, the area covered by Thio-S^+^ Aβ plaques, were presented in **b**, **d** (*n* = 9–10 per group, mean ± SD, *P < 0.05, **P < 0.01, Student’s *t* test), Scale bar = 600 µm. **e–h** Increased insoluble human Aβ40 and Aβ42 levels in CTX and HIP as well as soluble human Aβ42 in CTX of VPS35^CX3CR1^:5XFAD mice, as compared with those of Ctrl:5XFAD mice (*n* = 3 male per group, mean ± SD, **P < 0.01, Student’s *t* test)

It is known that the β-amyloid deposition in 5XFAD mice is a key pathology for AD development [21]. We thus first examined brain Aβ deposition by Thio-S staining in both groups of brain sections. In line with a previous report [31], few Thio-S positive Aβ plaques were detected in 3-MO Ctrl:5xFAD brain (Fig. 1a, b). Interestingly, the Thio-S positive Aβ plaques were significantly increased in multiple brain regions, including cortex (CTX), hippocampus (HIP), subiculum (SUB) and thalamus (TH) of VPS35^CX3CR1^:5xFAD mice (in both males and females) (Fig. 1a–d). These results suggest an increased Aβ deposition by microglial VPS35 deficiency in 5XFAD brain.

It’s worth noting that the Thio-S positive plaque is the compact insoluble Aβ. To determine whether the soluble/ insoluble human Aβ and mouse Aβ levels are altered in 5XFAD brain by microglial VPS35 deficiency, we measured their levels by ELISA analysis. In line with the increased Aβ deposition, insoluble guanidine fraction of human Aβ40 and Aβ42 levels in cortex and hippocampus as well as the soluble PBS fraction of human Aβ42 levels in cortex were elevated in VPS35^CX3CR1^:5xFAD mice (Fig. 1e–h). No difference was detected with the endogenous mouse Aβ40 levels in both cortex and hippocampus (Additional file 1: Fig. S2a, b). Together, these results suggest the Aβ deposition and Aβ levels were increased in VPS35^CX3CR1^:5xFAD brain, indicating that the microglial VPS35 deficiency exacerbates Aβ pathology in 5XFAD mice.

**Fig. 2:**
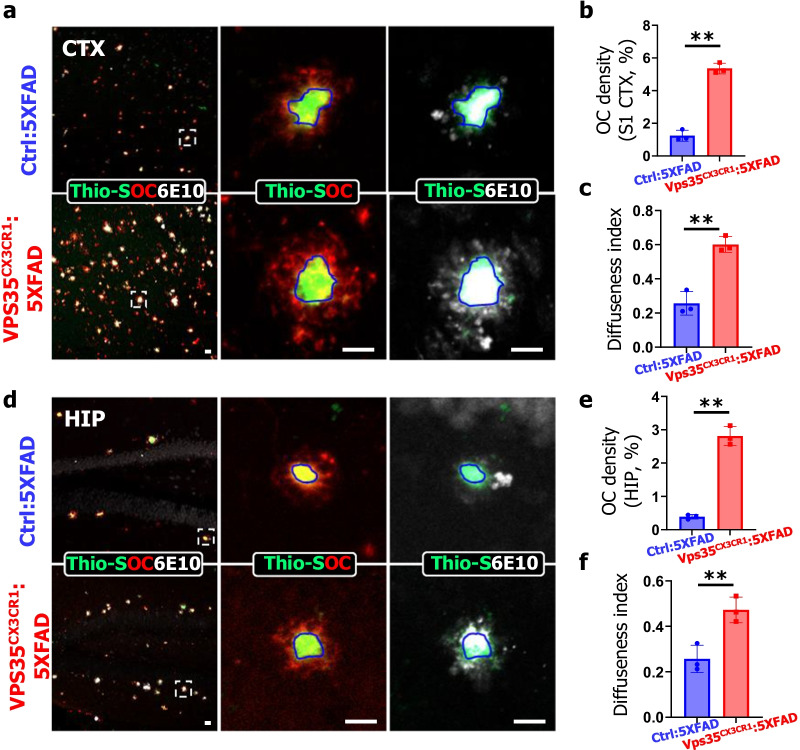
Increased Aβ oligomers and reduced plaque consolidation in microglial VPS35 deficient 5XFAD brain. **a**, **d** Representative images of Thio-S (a marker for compact fibrillar Aβ, green), OC (a marker for oligomeric Aβ, red) and 6E10 (for fibrillar Aβ) staining in the CTX (cortex) (**a**–**c**) and HIP (hippocampus) (**d**–**f**) of Ctrl:5XFAD and VPS35^CX3CR1^:5XFAD male mice. High-magnification images, marked by dashed squares, were shown in right panels. Scale bars, 10 μm. (**b**, **c**, **e**, **f)** Quantifications of OC covered area (**b**, **e**) and diffuseness index (**c**, **f**) in the CTX (**b**, **c**) and HIP (**e**, **f**). The diffuseness index was defined as: (Area_6E10_ − Area_Thio-S_)/ Area_6E10_. (*n* = 3 female mice per group, mean ± SD, **P < 0.01, Student’s *t* test)

We further examined Aβ plaque compaction by co-immunohistochemical staining analysis with Thio-S (a marker of compact amyloid), OC (a maker for diffused pro-fibrillar amyloid oligomers), and 6E10 antibody (a marker for total amyloid staining) (Fig. 2a, d). Remarkably, the portion of OC covered area was significantly increased in VPS35^CX3CR1^:5xFAD brain in both cortex and hippocampus, suggesting a reduction in plaque compaction by microglial VPS35 deletion (Fig. 2b, e). In addition, the assessment of diffuseness index (Diffuseness index = (Area_6E10_ − Area_Thio-S_)/ Area_6E10_) also showed an increase of the plaque diffuseness in the cortex and hippocampus of VPS35^CX3CR1^:5xFAD mice as compared with that of Ctrl:5xFAD mice (Fig. 2c, f). In aggregates, these results suggest that the lack of microglial VPS35 enhances the diffuse amyloid deposits and reduces plaque compaction.

### Elevated dystrophic neurites and reactive astrogliosis in microglial VPS35 deficient 5XFAD brain

**Fig. 3:**
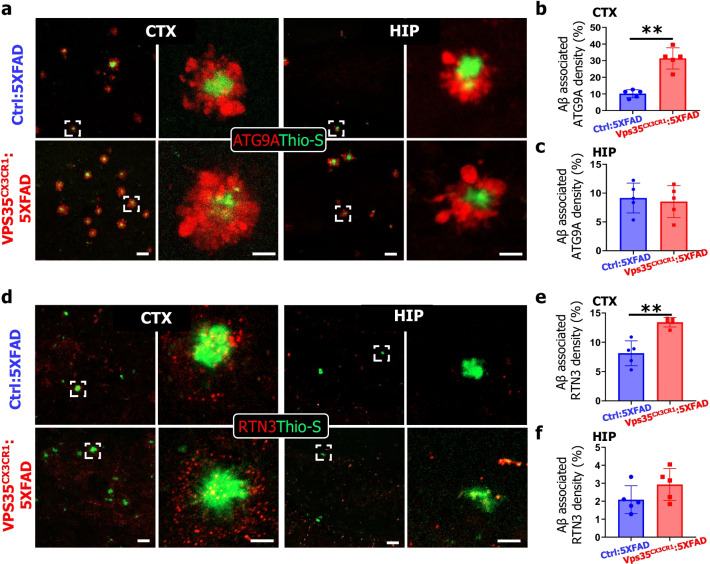
Elevated dystrophic neurites in microglial VPS35 deficient 5XFAD brain. **a** Representative image for ATG9A (red) and Thio-S (green) immunostaining in the CTX (cortex) and HIP (hippocampus). Scale bar = 10 μm. **b, c** Quantifications of Aβ associated ATG9A^+^ fluorescence density per plaque in CTX (**b**) and HIP (**c**) (*n* = 5 male mice per group, mean ± SD, **P < 0.01, Student’s *t* test). **d** Representative images for RTN3 (red) and Thio-S (green) immunostaining in the CTX and HIP. Scale bars, 10 μm. **e**, **f** Quantifications of Aβ associated RTN3 fluorescence density per plaque in the CTX (**e**) and HIP (**f**) (*n* = 5 male mice per group, mean ± SD, **P < 0.01, Student’s *t* test)

The extracellular deposition of Aβ is often surrounded with swollen, dystrophic dendrites or axons, so called dystrophic neurites, which is recognized as one of pathological features in AD patients and animal models [32, 33]. Given the increased Aβ deposition in microglial VPS35 deficient 5XFAD brain, we asked whether the plaque-associated dystrophic neurites were also elevated**.** Both ATG9A (a protein of pre-autophagosome) and RTN3 (a protein of the tubular endoplasmic reticulum, ER) are markers for dystrophic neurites, which were highly enriched at the inner layer and outer layer of the beginning formation site of deposited plaque, respectively [33–35]. Co-immunostaining analyses using the antibodies against the ATG9A with Thio-S showed increased plaque-associated ATG9A^+^ dystrophic neurites’ density in VPS35^CX3CR1^:5xFAD cortex, but not in the hippocampus (Fig. 3a–c). The increased dystrophic neurites were further confirmed by Thio-S co-immunostaining with antibody against RNT3 (Fig. 3d–f). These results demonstrate the increased amyloid plaques with microglial VPS35 deficiency accompanied by the increased neural toxicity, suggesting microglial VPS35's function in preventing both Aβ accumulation and DN formation.

The extracellular Aβ deposition is also often associated GFAP positive (+) reactive astrocytes in brains of AD patients and animal models [21, 24, 36]. We thus performed co-immunostaining analysis using antibodies against GFAP and ApoE, an AD risk gene that is also highly expressed in astrocytes [37, 38]. Indeed, a marked increase in GFAP^+^ astrocytes was detected in *VPS35*^*CX3CR1*^*:5xFAD* cortex (Additional file 1: Fig. S3a, b). However, the plaque associated GFAP^+^ astrocytes (or the number of astrocytes per plaque) in the *VPS35*^*CX3CR1*^*:5xFAD* cortex were comparable to that of control: 5XFAD (Additional file 1: Fig. S3d). Notice that the ApoE^+^ astrocytes and the ApoE^+^Thio-S^+^ plaques were both elevated in the *VPS35*^*CX3CR1*^*:5xFAD* cortex (Additional file 1: Fig. S3a, c, e). These results suggest an increased reactive astrogliosis in *VPS35*^*CX3CR1*^*:5xFAD* cortex, in line with the view for the elevated Aβ deposition.

### Cognition decline, but not depression, in young adult microglial VPS35 deficient 5XFAD mice

**Fig. 4:**
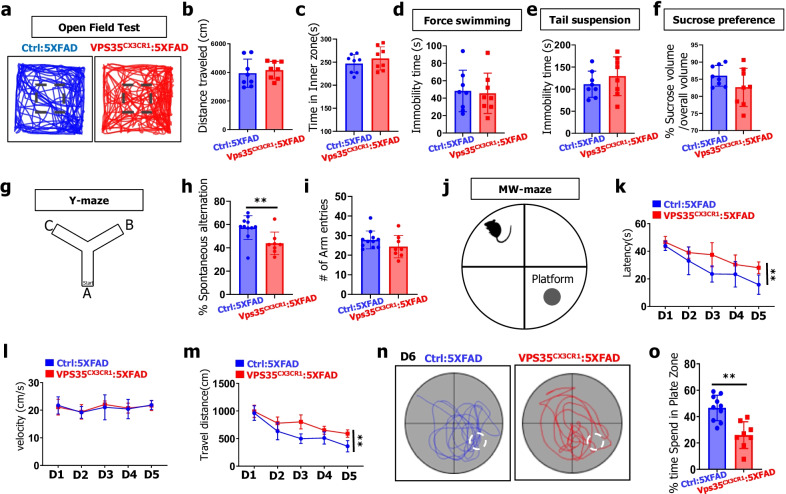
Enhanced cognitive dysfunction, but not depression, in young adult microglial VPS35 deficient 5XFAD mice. **a**–**c** Open field test (OFT). Representative traces of mouse movement path in Ctrl:5XFAD mice and VPS35^CX3CR1^:5XFAD mice (3-MO) (**a**). Total distance traveled (**b**) and time spent inner zone (**c**) were shown (*n* = 6 male mice per group, mean ± SD). **d**–**f** Immobility time in forced swimming test (FST), tail suspension test (TST), and sucrose preference by sucrose preference test (SPT) were shown. The sucrose preference = consumed sucrose/total consumed liquid. (*n* = 6 male mice per group, mean ± SD). **g**–**i** Y-maze test. Schematic diagram (**g**), and reduced spontaneous alternations, but no the number of arm entries, in VPS35^CX3CR1^:5XFAD mice were shown (*n* = 6 male mice per group, mean ± SD, **P < 0.01). **J–m** Morris water maze (MWM) assay. Ctrl:5XFAD (*n* = 6) and VPS35^CX3CR1^:5XFAD (*n* = 6) male mice were examined. The VPS35^CX3CR1^:5XFAD mice exhibited increases in the latency and travel distance to the hidden platform (mean ± SD, *P < 0.05). **n–o** Motion trajectory in Ctrl:5XFAD mice and VPS35^CX3CR1^:5XFAD mice after removing hidden platform at day 6 (**n**), and quantification of the time spend in the hidden platform zone (**o**). (Mean ± SD, **P < 0.01, Student’s *t* test)

AD is clinically characterized by the decline of cognitive function, and accompanied with negative affective symptoms, such as depression [39]. 5XFAD mice display similar behavior changes, including learning and memory deficit and depression, in an age dependent manner [40, 41]. We thus asked whether microglial VPS35 deficiency in 5XFAD mice affects these behavior phenotypes. First, we assessed mouse anxiety and depression-like behaviors by use of a combination of open field test (OFT) (Fig. 4a), force swimming test (FST) (Fig. 4d), tail suspension test (TST) (Fig. 4e), and sucrose preference test (SPT) (Fig. 4f). Little to no differences were detected between the two groups of mice (*VPS35*^*CX3CR1*^*:5xFAD* vs Ctrl: 5XFAD) in viewing their total traveled distances and time spent in the inner zone during OFT (Fig. 4b, c), the immobility times during FST (Fig. 4d), and TST (Fig. 4e), and the sucrose volumes by the SPT (Fig. 4f). These results suggest that microglial VPS35 deficiency plays little role in precipitation of the anxiety and depression-like behaviors in 5XFAD mice. We second evaluated cognitive function in both growth mice by use of Y-maze test (to assess spatial working memory) (Fig. 4g) [42]. Interestingly, VPS35^CX3CR1^:5xFAD mice exhibited a reduced spontaneous alternation (Fig. 4h), but not the number of arm entries (Fig. 4i), as compared with those in ctrl:5xFAD mice, during Y-maze test, suggesting a deficit on spatial working memory. Third, we subjected both groups of mice (VPS35^CX3CR1^:5xFAD and ctrl:5xFAD) to the Morris water maze (MWM) to access their spatial learning and memory [43]. Intriguingly, the VPS35^CX3CR1^:5xFAD mice showed increased latency and the travel distance to find the escape platform, without changes in the velocity during the 5-day training phase (Fig. 4j–m), and decreased time in target zone at the 6^th^ day probe phase (Fig. 4n, o). Taken together, these results suggest that microglial VPS35 deficiency exacerbates cognitive disfunction (spatial learning and memory), but not anxiety- or depression-like behaviors, in VPS35^CX3CR1^:5xFAD mice.

### Reduced plaque-associated myeloid cells in microglial VPS35 deficient 5XFAD brain

**Fig. 5:**
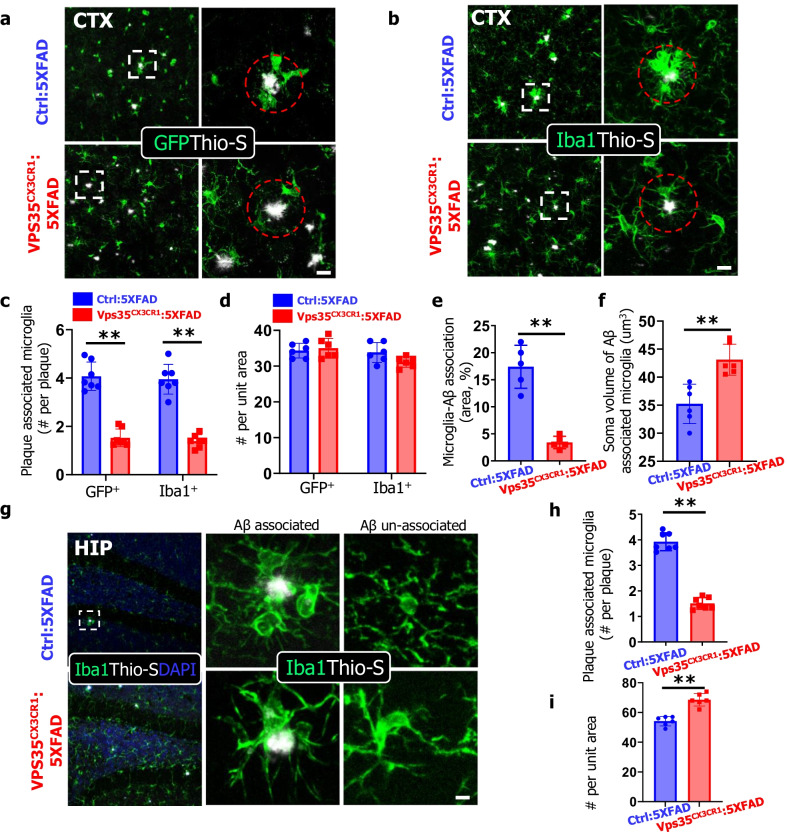
Reduced plaque-associated myeloid cells in microglial VPS35 deficient 5XFAD brain. **a, b** Representative images for GFP (green) (**a**), Iba1 (**b**), and Thio-S (white) immunostaining in the CTX (cortex). High-magnification images, marked by dashed squares, were shown in right panels. The plaque associated microglia were defined by number of microglia in a plaque centered circle within 50 μm in diameter (marked by dashed red circles). Scale bars, 10 μm. **c–f** Quantification analyses of data in **a**, **b**. The plaque associated microglia (number per plaque) by co-immunostaining GFP or Iba1 with Thio-S were shown in **c**; the microglial density (number per unit area) (319.45 μm^2^) was shown in **d**; the microglia-Aβ association by assessing the percentage of covered area was shown in **e**; and the soma volume of Aβ associated microglia measured by Imaris software was shown in **f** (*n* = 5–6 male mice per group, mean ± SD, **P < 0.01, Student’s *t* test). **g** Representative images for Iba1 (green) and Thio-S (white) immunostaining in the HIP. High-magnification images were shown in right panels. Scale bar, 10 μm. **h–i** Quantitative analysis of data in **g**. The plaque associated microglia (number per plaque) (**h**) and microglial density (number per unit area) (**i**) were shown (*n* = 6 male mice per group, mean ± SD, **P < 0.01, Student’s *t* test)

To understand how microglial VPS35 deficiency induces more Aβ deposition, Aβ associated brain pathology, and cognitive dysfunction in 5XFAD mice, we examined microglial cell phenotypes in response to Aβ in the 5XFAD brain. Microglia are known to be rapidly recruited to the newly formed amyloid plaques [44]. We thus carried out co-immunostaining analysis using antibodies against GFP (a marker for microglia in Cx3Cr1–CreER mice) with Thio-S in brain sections from control and microglial VPS35 deficient 5XFAD mice. No difference in the GFP marked microglial cell density in cortex was detected between control and microglial VPS35 deficient 5XFAD mice (Fig. 5a, d). However, in the Aβ associated area (within a diameter of 50 um of Thio-S^+^ core, indicated as red dash circle), the clustered GFP^+^ microglia were abundant in the Ctrl:5xFAD cortex, but marked reduced in the VPS35^CX3CR1^:5xFAD mice (Fig. 5a). Quantification analysis showed a significant reduction in Aβ associated microglial cell number in the microglial VPS35 deficient 5XFAD mice, as compared with those of control:5XFAD mice (Fig. 5c). This observation was further verified by co-immunostaining analysis with antibody against Iba1 (a marker of microglia) with Thio-S (Fig. 5b, e, f). In addition, the percentage of microglial cell covering Aβ area was lower in the VPS35^CX3CR1^:5xFAD cortex (Fig. 5e), in line with the view of reduced Aβ compaction. Moreover, examining microglial cell morphology showed larger soma volume in Aβ associated and un-associated microglia and more processes in Aβ un-associated microglia in VPS35^CX3CR1^:5xFAD cortex, as compared with those of control:5XFAD mice (Fig. 5f, Additional file 1: Fig. S4a–c). The similar reduction in Aβ-associated microglia was also detected in the VPS35^CX3CR1^:5xFAD hippocampus (Fig. 5g–i). However, in contrast from the result in the mutant cortex, the Iba1^+^ cell density was higher in the hippocampus of VPS35^CX3CR1^:5xFAD than that of control: 5XFAD (Fig. 5g, i), in agreement with our previous report [15]. Taken together, these results suggest that the microglial VPS35 is necessary for Aβ-recruitment of microglia in young adult 5XFAD mice.

### Decreased DAM in microglial VPS35 deficient 5XFAD brain

**Fig. 6:**
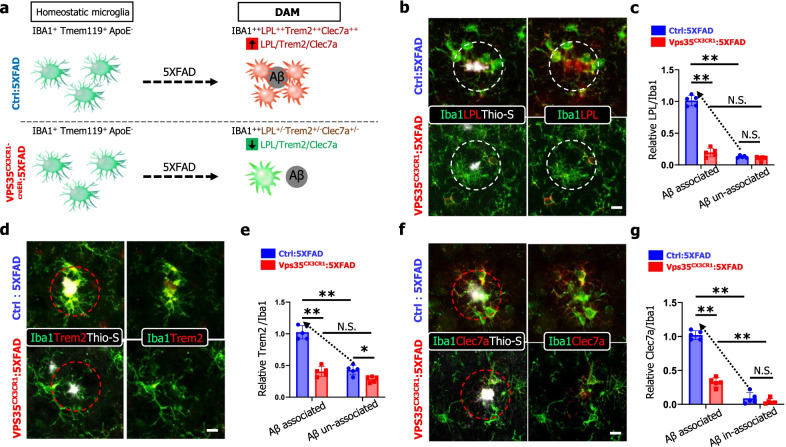
Decreased DAM in microglial VPS35 deficient 5XFAD brain. **a **Schematic diagram of Aβ associated microglia or DAM (disease associated microglia), and markers for DAM; and summary of the results about DAMs in control and microglial VPS35 deficient 5XFAD brain. **b, c** Representative images for Iba1 (green), LPL (red), and Thio-S (gray) immunostaining (**b**), and quantification of relative LPL fluorescence intensity in Aβ associated and Aβ un-associated microglia (**c**). **d, e** Representative images for Iba1 (green), Trem2 (red) and Thio-S (gray) immunostaining (**d**), and quantification of relative Trem2 fluorescence intensity in Aβ associated and Aβ un-associated microglia (**e**). **f, g** Representative images for Iba1 (green), Clec7a (red) and Thio-S (gray) immunostaining (**f**), and quantification of relative Clec7a fluorescence intensity in Aβ associated and Aβ un-associated microglia (**g**). Scale bars, 10 μm. Data in **c**, **e**, and **g** are presented as mean ± SD (*n* = 5 male mice per group, *P < 0.05, **P < 0.01, N.S., *P* > 0.05, Student’s *t* test)

It is of interest to note that the Aβ associated microglia exhibit molecular features of disease-associated microglia (DAM) by recent scRNA-seq analysis [7]. The reduction in Aβ associated microglia in microglial VPS35 deficient 5XFAD brain leads to the speculation for microglial VPS35's function in DAM development. To test this view, we first performed co-immunostaining analysis using antibodies against DAM markers, such as LPL (Lipoprotein Lipase), Clec7a (C-Type Lectin Domain Containing 7A), and Trem2 (Triggering Receptor Expressed on Myeloid Cells 2) with Iba1 (to label both homeostatic microglia and DAM) and Thio-S (to mark Aβ plaques) (Fig. 6a). Notice that in the Ctrl:5xFAD cortex, the Thio-S^+^ plaques were surrounded with microglia that were positive (+) for both LPL and Iba1 (indicated by white dashed circle), whereas plaque un-associated microglia were Iba1^+^, but LPL^−^ (Fig. 6b, c), supporting the view that the plaque associated microglia exhibit features of DAM [7, 45]. In contrast, in VPS35^CX3CR1^:5xFAD mice, the Thio-S^+^ plaque associated Iba1^+^ microglia had little expression of LPL (Fig. 6b, c), suggesting a reduction of LPL^+^ DAM in the microglial VPS35 deficient 5XFAD cortex. This view was further supported by the observations that the plaque associated microglia in the Vps35 mutant 5XFAD cortex also expressed little Clec7a and Trem2, two other markers of DAM (Fig. 6d–g).

**Fig. 7:**
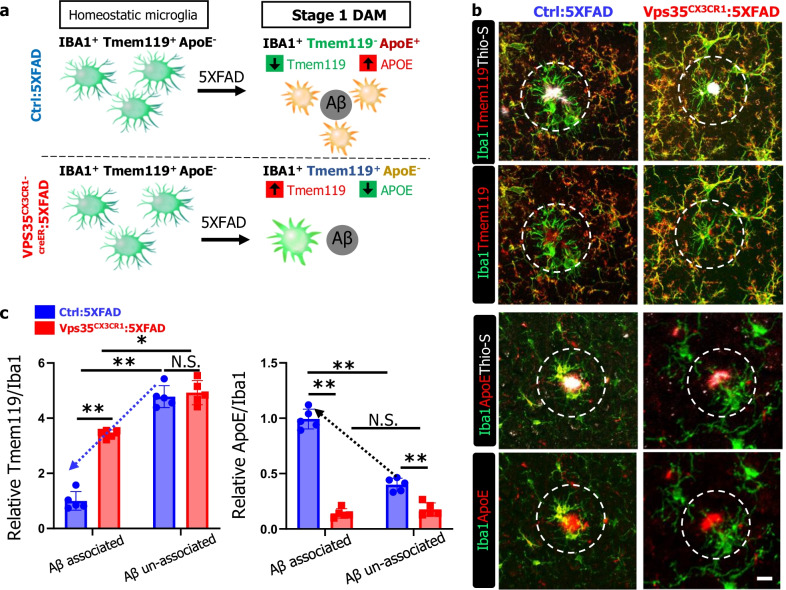
Decreased stage 1 DAM in microglial VPS35 deficient 5XFAD brain. **a **Schematic diagram of Aβ associated microglia in microglial VPS35 deficient 5XFAD brain leads to the reduction in Stage1 DAM activation. **b** Representative images for Tmem119, ApoE with Iba1 (green) and Thio-S (gray) immunostaining. Scale bar = 10 μm. **c** Quantification of relative Tmem119 and ApoE fluorescence intensity in Aβ associated and Aβ un-associated microglia (*n* = 5 male mice per group, mean ± SD, *P < 0.05, **P < 0.01, N.S., *P* > 0.05, Student’s *t* test)

DAM has been classified as stage I and stage II, based on single cell transcriptomic analysis that uncovers different marker gene expression profile [7]. The LPL, Clec7a, and Trem2 are all markers for stage II DAM (Fig. 7a) [7, 46]. To determine whether microglial VPS35 regulates stage I DAM development, we carried out additional co-immunostaining analysis using antibodies against Tmem119 and ApoE (Fig. 7a), because Tmem119, a marker for homeostatic microglia [47], is reduced in stage I DAM [7]; and ApoE, which is highly expressed in astrocytes and little in the homeostatic microglia, is upregulated in stage I DAM [7, 48, 49]. Indeed, the plaque associated Iba1^+^ microglia (indicated by white dash circle) had nearly un-detectable level of Tmem119, or at a much lower level than those of plaque un-associated Iba1^+^ microglia (likely to be homeostatic microglia), in the ctrl:5xFAD cortex (Fig. 7b), in line with the view for a reduced Tmem119 in DAM. Interestingly, in VPS35^CX3CR1^:5xFAD cortex, Tmem119 remained positive in the Thio-S plaque associated Iba1^+^ microglia, with a slight reduction as compared with those of plaque un-associated Iba1^+^ microglia (Fig. 7b, c). In addition, APOE was positive not only in Thio-S^+^ plaques, but also in plaque associated Iba1^+^ microglia, in the ctrl:5xFAD cortex (Fig. 7b). However, in VPS35^CX3CR1^:5xFAD cortex, APOE was detected in Thio-S^+^ plaques, but little to no positive signal in the plaque associated Iba1^+^ microglia (Fig. 7b, c). Together, these results suggest an impairment in DAM (at both stages I and II) formation in microglial VPS35 deficient 5XFAD cortex.

### Impaired phagocytosis of Aβ42, but not pHrodo beads, in microglial VPS35 deficient cortex

**Fig. 8:**
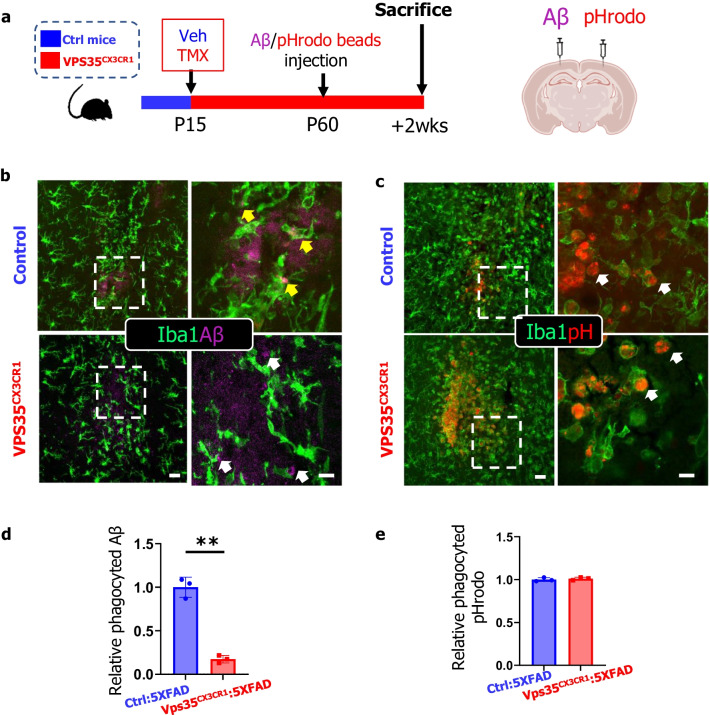
Impaired phagocytosis of Aβ, but not pHrodo beads, in microglial VPS35 deficient brain. **a **Schematic diagram of Aβ42 and pHrodo beads injection to the cortex at P60, which were sacrificed at 2 weeks after injection. **b, c** Representative images for Aβ42 (purple) and pHrodo beads (red) with Iba1 (green) immunostaining. Scale bar = 10 μm. **d, e** Quantification of fluorescence intensity of phagocyted Aβ42 and pHrodo beads (*n* = 3 male mice per group, mean ± SD, **P < 0.01, Student’s *t* test)

Notice that DAM cells express abundant genes that are involved in phagocytic and lipid metabolism [7, 50, 51]. Given the reduced DAM in microglial VPS35 deficient 5XFAD brain, we speculate that the DAM microglia may play a critical role in regulating Aβ phagocytosis and clearance, which is impaired by microglial VPS35 deficiency. To test this view, Aβ42 and pHrodo Red zymosan bioparticles (as a control) were injected into the cortex of control (Vps35^CX3Cr1^ mice with vehicle injection) and Vps35^CX3Cr1^ mice (with tamoxifen injection), as illustrated in Fig. 8a. Co-immunostaining analysis showed Iba1^+^ microglial cell internalized Aβ42-488, which was obvious in the control cortex, but little to un-detectable in the VPS35^CX3CR1^ cortex (Fig. 8b, d), suggesting an impairment in Aβ42 uptake in the mutant mice. This effect appears to be specific, because a comparable level of pHrodo Red zymosan bioparticles in Vps35 deficient microglia to those of control microglia was observed (Fig. 8c, e).

### Coupling of microglial Aβ phagocytosis with DAM formation

**Fig. 9:**
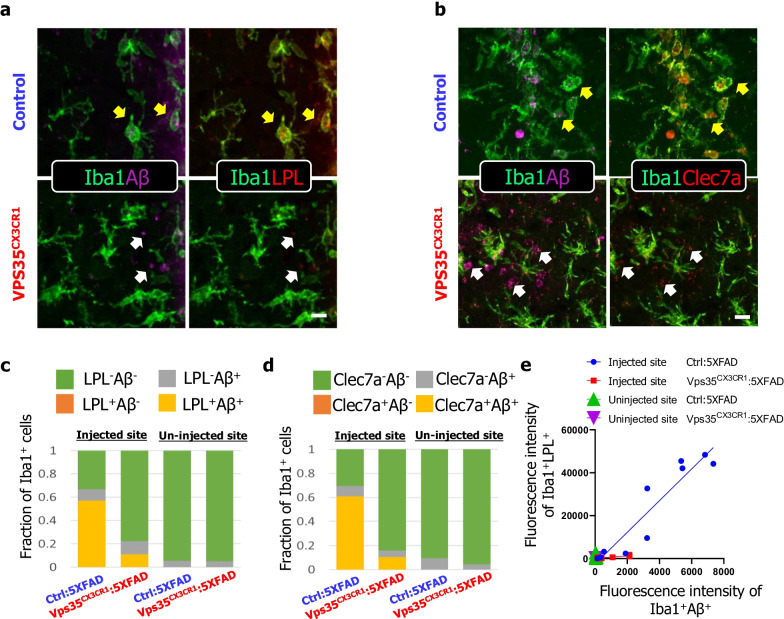
Coupling of microglial Aβ42 phagocytosis with DAM formation. **a **Representative image for Aβ42 (purple), LPL (red) with Iba1 (green) immunostaining in the cortex after Aβ42 injection. Scale bar = 10 μm. **b** Representative images for Aβ42 (purple), Clec7a (red) with Iba1 (green) immunostaining in the cortex after Aβ42 injection. Scale bar = 10 μm. **c** Bar plot showing the fraction of IBA1^+^ cells in the injected site and un-injected site (*n* = 3 male mice per group). **d** Quantification of the correlation between the fluorescence intensity of Iba1^+^Aβ^+^ and fluorescence intensity of Iba1^+^LPL^+^

The spatial, temporal, and molecular associations of DAM with Aβ42 phagocytosis led us to ask whether Aβ42 uptake could induce DAM formation. To test this view, we carried out additional co-immunostaining analysis using antibodies against Iba1 and DAM markers (LPL and Clec7a) in Aβ42 injected brain samples. Indeed, while Aβ42 was internalized by the microglia, these microglia were also positive for LPL and Clec7a in the control brain samples injected with Aβ42 (Fig. 9a, b). In contrast, in VPS35^CX3CR1^ mice, little Aβ42 was phagocytosed by microglia, and the Iba1^+^LPL^+^ or Iba1^+^Clec7a^+^ DAM cells were also little or undetectable (Fig. 9c–e). These results demonstrate a tight association between Aβ42 phagocytosis and DAM formation, and in line with the view for DAM to be responsible for Aβ uptake; and microglial cell up-taken Aβ may also induce DAM marker gene expression and DAM development.

## Discussion

**Fig. 10:**
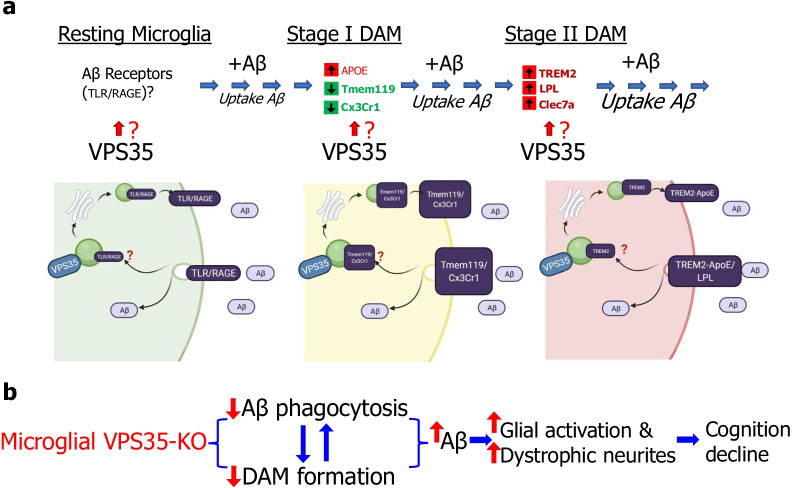
Model of microglial VPS35’s function in Aβ pathology and AD development. **a **Schematic diagram of microglial VPS35 in DAM development at multiple steps by regulate membrane Aβ receptors. **b** Microglial VPS35 deficiency precipitates Aβ pathology and AD development, likely by impair DAM development and Aβ phagocytosis, inducing increased Aβ, glial activation and dystrophic neurites formation, and finally leading cognition decline

The dysfunctional VPS35/retromer is believed to be a risk factor for AD, because it is reduced in AD patients' hippocampus. This view is further supported by the observation that Vps35 haploinsufficiency in Tg2576 AD animal model enhances AD neuropathology. VPS35/retromer is expressed in neurons and glial cells in the brain. While neuronal VPS35 is critical to prevent neurodegeneration during development, the functions of microglial VPS35 in AD development are beginning to be un-folded. Here, we provide evidence for microglial VPS35-deficiency to enhance AD-relevant pathology in 5XFAD mouse model. Our studies lead us to propose a working hypothesis depicted in Fig. 10, in which, microglial VPS35 plays a key role in promoting Aβ uptake and DAM development, uncovering a mechanism underlying microglial VPS35-loss in AD development.

VPS35 expression is reduced not only in AD patients' hippocampus [14], but also in microglia isolated from AD patients’ brain [20], implicating microglial VPS35 deficiency in AD pathogenesis. It is known that the cognitive dysfunction, Aβ accumulation, and dystrophic neurites formation were the hallmarks of AD in various AD mouse models [52, 53]. Interestingly, microglial VPS35 loss in 5xFAD mice, a well-characterized AD animal model, increases Aβ associated brain pathology, including Aβ insoluble, fibrillar, and plaque forms (Figs. 1, 2), dystrophic neurites (Fig. 3), reactive astrocytes (Additional file 1: Fig. S3) and worse the learning and memory behaviors (Fig. 4). In addition, these observations thus support the view for microglial VPS35 deficiency to precipitate AD pathology. This view is also in line with a previous report that mice with specific microglial VPS35 depletion exhibit microglial activation selectively in the hippocampus and impairment in adult hippocampal neurogenesis [15].

How does microglial VPS35 deficiency increase Aβ pathology? It is of interest to note that the Aβ plaques are surrounded with a special subtype of microglia, so called DAM, which exhibit distinct molecular features, such as increases in APOE, LPL, TREM2, and Clec7a, and decreases in Cx3Cr1, P2ry12, and TMEM119 [7, 51]. Microglial VPS35 loss in 5XFAD brain results in reduced microglia that surround the Aβ plaque, but not the microglia un-associated with Aβ plaque (Fig. 5), and decreased expressions of LPL, Clec7a, TREM2, and ApoE, but increased Tmem119 in microglia that surround Aβ plaques (Figs. 6, 7), supporting the view for an impaired DAM development in microglial VPS35 deficient brain.

It is known that DAM is induced by multiple pathological stimuli, including Aβ, stroke injury, and other neurodegenerative disease, such as ALS [7, 54]. DAM development includes two stages, I and II, which are marked with distinctive proteins [7]. Interestingly, microglial VPS35 loss reduces both stages of DAM in 5XFAD brain (Figs. 6, 7), supporting a critical role of microglial VPS35 in Aβ induced DAM formation. This view was further supported by the observations that Aβ injection into the brain only induce DAM in control, but not microglial VPS35 knockout, mice (Figs. 8, 9). Intriguingly, the Aβ induction of DAM is tightly coupled with the microglial uptake of Aβ (Fig. 9); and both events are impaired by microglial VPS35-loss (Figs. 8, 9). In light of these observations and literature reports, we speculate that the microglial VPS35 plays important roles in DAM development at multiple steps, including promoting the initial microglial Aβ phagocytosis, likely by regulating the trafficking and functions of Aβ receptors, such as TLR and RAGE (Fig. 10a), and promoting stage I and II DAM development, likely by regulating APOE’s expression and TREM2 receptor’s trafficking, respectively (Fig. 10a). Notice that both APOE and TREM2 bind to Aβ and promote Aβ phagocytosis [55–57]; and TREM2 is reported to be a cargo protein of VPS35/retromer [16], in line with the working hypothesis (Fig. 10a). It is also noteworthy that not only Aβ induced DAM is impaired in microglial VPS35 KO 5XFAD mice, the ischemic stroke induced DAM was also diminished in microglial VPS35 KO cortex [19]. In addition, microglial VPS35-loss induced microglial cell activation in the hippocampus and reduced adult neurogenesis may also be involved in AD development (Fig. 10b) [15]. However, this view requires further investigation.

## Conclusions

In summary, this study suggests that microglial VPS35 deficiency enhances Aβ pathology and accelerates cognitive decline in 5XFAD mice, an AD animal model, supporting the view for VPS35 deficiency as a risk factor for AD development. Mechanistically, we provide evidence that microglial VPS35 deficiency impairs DAM development and DAM mediated Aβ phagocytosis, revealing a possible cellular mechanism underlying the increased Aβ pathology. Further studies are necessary to investigate the molecular mechanisms by which microglial VPS35 promotes DAM development and Aβ pathology.

## Supplementary Information

**Additional file 1: Figure S1.** Mouse breeding and experimental protocol. a Breeding schematic to obtain *VPS35*^*f/f*^:*CX3CR1*^*Cre−ER*^*:5xFAD* mice. b Schematic illustrating TMX injection and behavioral testing timeline. Tamoxifen (100 mg/kg) was administered (i.p.) into *VPS35*^*f/f*^:*CX3CR1*^*Cre−ER*^*:5xFAD* mice [as mutants (VPS35^CX3CR1^:5XFAD)]. In addition, tamoxifen was injected to *VPS35*^*f/f*^*:5xFAD* or vehicle (Corn oil) was injected to *VPS35*^*f/f*^:*CX3CR1*^*Cre−ER*^*:5xFAD* mice [as controls (Ctrl:5XFAD)]. c Double immunostaining analysis for Iba1 (green) and VPS35 (Red) immunostaining. Scale bar = 10 μm. d Quantitative analysis of relative microglial VPS35 intensity compared with Ctrl: 5xFAD mice. (*n* = 3 per group, mean ± SD, **, *P* < 0.01, Student’s *t* test) e Weight analysis was conducted in 3 months Ctrl:5XFAD and VPS35^CX3CR1^:5XFAD mice. (*n* = 7 per group, mean ± SD). **Figure S2.** No difference detected in endogenous mouse Aβ40 levels in microglial VPS35 deficient 5XFAD brain. a, b Expression of endogenous mouse Aβ40 level (*n* = 3 per group, mean ± SD, N.S., P > 0.05, Student’s *t* test). **Figure S3. **Increased reactive astrocytes in microglial VPS35 deficient 5XFAD brain. a Co-immunostaining of GFAP (green), ApoE (red) and Thio-S (gray) in the cortex of Ctrl:5XFAD and VPS35^CX3CR1^:5XFAD mice. b–e Quantification of GFAP^+^ cell density, ApoE^+^ cell density, average ApoE fluorescence intensity per astrocyte and relative ApoE^+^Thio-S^+^ density in the cortex of Ctrl:5XFAD and VPS35^CX3CR1^:5XFAD mice (*n* = 5 per group, mean ± SD, **, *P* < 0.01, Student’s *t* test). **Figure S4**. Increased branch number and soma volume in microglial VPS35 deficient 5XFAD brain. a Representative image for Aβ un-associated microglia and 3D reconstitution was performed with Imaris. Scale bar = 10 μm. b, c Quantitative analysis branch number and soma volume of Aβ un-associated microglia in Ctrl:5XFAD and VPS35^CX3CR1^:5XFAD mice (*n* = 5 per group, mean ± SD, **, *P* < 0.01, Student’s *t* test).

## Data Availability

All data generated or analyzed during this study are included in the published article.

## Abbreviations

VPS35: Vacuolar sorting protein 35
AD: Alzheimer’s disease
cKO: Conditional knockout
Aβ: Amyloid-beta
DAM: Damage-associated microglia
PD: Parkinson’s disease
APP: Amyloid beta precursor protein
PSEN1: Presenilin 1
PFA: Paraformaldehyde
Thio-s: Thioflavin S
OFT: Open field test
FST: Force swimming test
TST: Tail suspension test
SPT: Sucrose preference test
MWM: Morris water maze
CTX: Cortex
HIP: Hippocampus
SUB: Subiculum
TH: Thalamus
DN: Dystrophic neurite
LPL: Lipoprotein lipase
Clec7a: C-type lectin domain containing 7A
Trem2: Triggering receptor expressed on myeloid cells 2
