# Supplementary figures and images for: Microglial VPS35 deficiency impairs Aβ phagocytosis and Aβ-induced disease-associated microglia, and enhances Aβ associated pathology
Source: J Neuroinflammation. 2022 Mar 2;19:61. doi: 10.1186/s12974-022-02422-0 (PMC8892702; doi:10.1186/s12974-022-02422-0)

**Fig. S1**

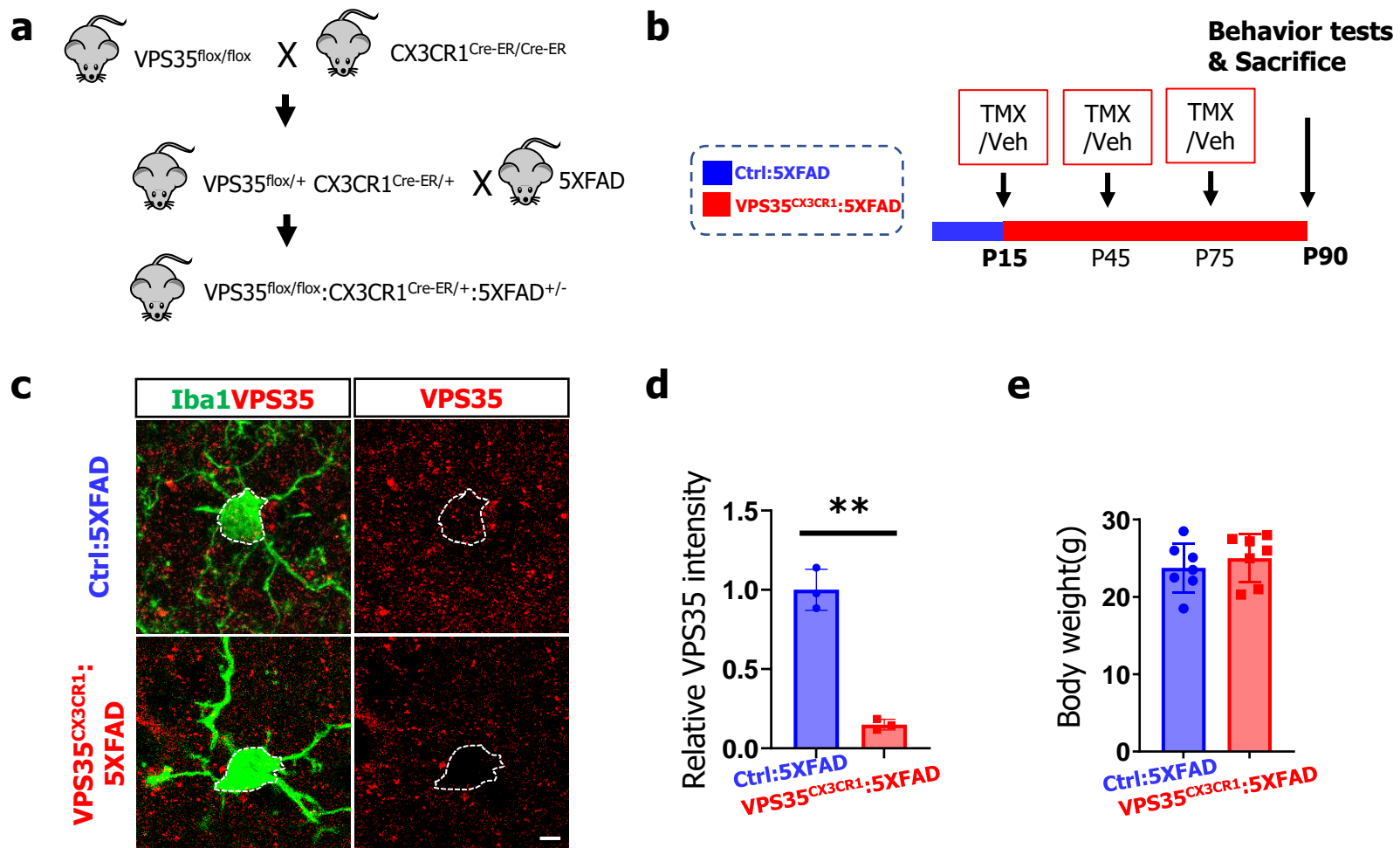

**Fig. S2**

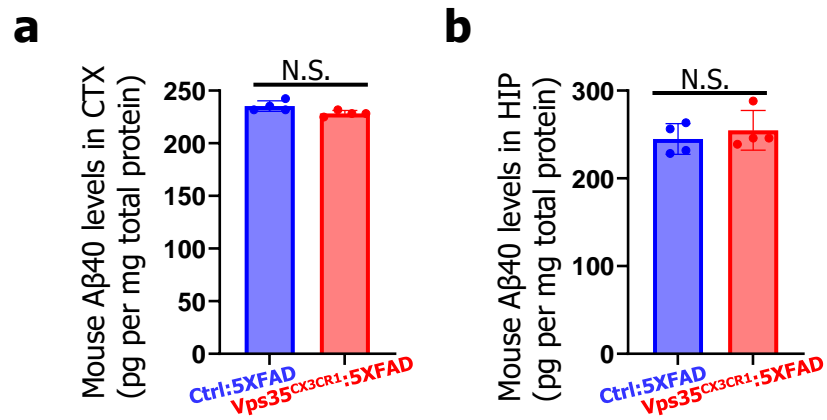

**Fig. S3**

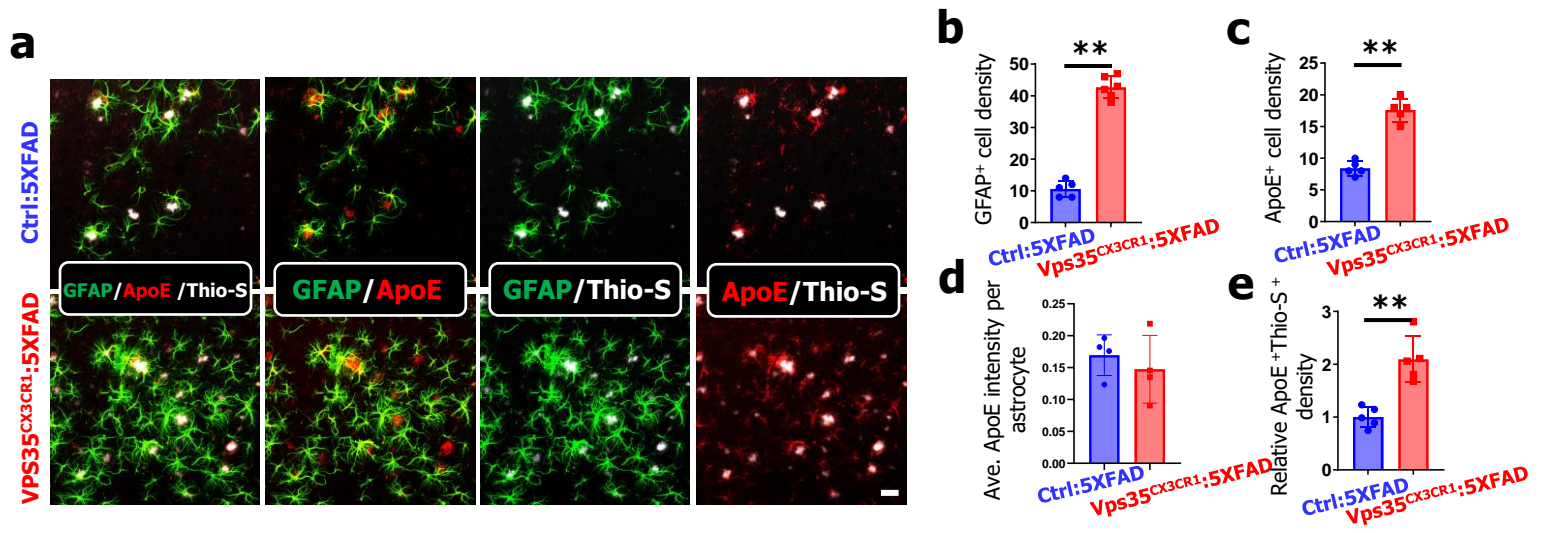

**Fig. S4**

**a**

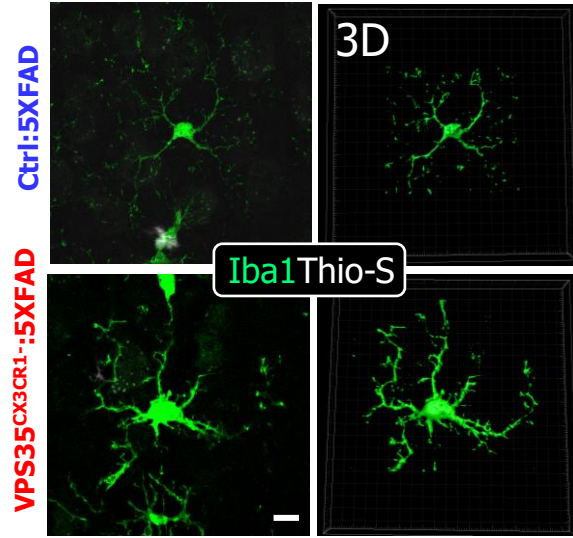

**b**

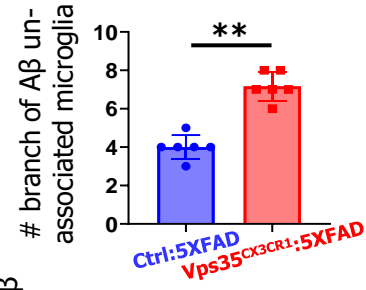

**c**

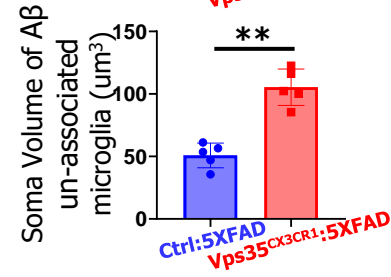

Supplement: Supplementary file 1 — Additional file 1: Figure S1. Mouse breeding and experimental protocol. a Breeding schematic to obtain VPS35f/f:CX3CR1Cre−ER:5xFAD mice. b Schematic illustrating TMX injection and behavioral testing timeline. Tamoxifen (100 mg/kg) was administered (i.p.) into VPS35f/f:CX3CR1Cre−ER:5xFAD mice [as mutants (VPS35CX3CR1:5XFAD)]. In addition, tamoxifen was injected to VPS35f/f:5xFAD or vehicle (Corn oil) was injected to VPS35f/f:CX3CR1Cre−ER:5xFAD mice [as controls (Ctrl:5XFAD)]. c Double immunostaining analysis for Iba1 (green) and VPS35 (Red) immunostaining. Scale bar = 10 μm. d Quantitative analysis of relative microglial VPS35 intensity compared with Ctrl: 5xFAD mice. (n = 3 per group, mean ± SD, **, P < 0.01, Student’s t test) e Weight analysis was conducted in 3 months Ctrl:5XFAD and VPS35CX3CR1:5XFAD mice. (n = 7 per group, mean ± SD). Figure S2. No difference detected in endogenous mouse Aβ40 levels in microglial VPS35 deficient 5XFAD brain. a, b Expression of endogenous mouse Aβ40 level (n = 3 per group, mean ± SD, N.S., P > 0.05, Student’s t test). Figure S3. Increased reactive astrocytes in microglial VPS35 deficient 5XFAD brain. a Co-immunostaining of GFAP (green), ApoE (red) and Thio-S (gray) in the cortex of Ctrl:5XFAD and VPS35CX3CR1:5XFAD mice. b–e Quantification of GFAP+ cell density, ApoE+ cell density, average ApoE fluorescence intensity per astrocyte and relative ApoE+Thio-S+ density in the cortex of Ctrl:5XFAD and VPS35CX3CR1:5XFAD mice (n = 5 per group, mean ± SD, **, P < 0.01, Student’s t test). Figure S4. Increased branch number and soma volume in microglial VPS35 deficient 5XFAD brain. a Representative image for Aβ un-associated microglia and 3D reconstitution was performed with Imaris. Scale bar = 10 μm. b, c Quantitative analysis branch number and soma volume of Aβ un-associated microglia in Ctrl:5XFAD and VPS35CX3CR1:5XFAD mice (n = 5 per group, mean ± SD, **, P < 0.01, Student’s t test). [file 12974_2022_2422_MOESM1_ESM.pdf]
